# Federated foundation models for psychiatry: a new paradigm for diagnosis, prognosis, and treatment of mood disorders

**DOI:** 10.3389/fpsyt.2026.1792429

**Published:** 2026-03-13

**Authors:** Maryam Ebrahimi, Rajeev Sahay, Bita Akram, Seyyedali Hosseinalipour

**Affiliations:** 1 Department of Mathematics, Sharif University of Technology, Tehran, Iran; 2 Department of Electrical and Computer Engineering, UC–San Diego, La Jolla, CA, United States; 3 Department of Computer Science, NC State University, Raleigh, NC, United States; 4 Center for Educational Informatics, NC State University, Raleigh, NC, United States; 5 Department of Electrical Engineering, University at Buffalo–SUNY, Buffalo, NY, United States; 6 Institute for Artificial Intelligence and Data Science (IAD), University at Buffalo–SUNY, Buffalo, NY, United States

**Keywords:** federated learning, foundation models, mental health, multimodal multitask learning, psychiatry

## Abstract

Multimodal Multitask Federated Foundation Models (M3T-FedFMs) represent a new frontier in artificial intelligence (AI), enabling integration of diverse data modalities and multitask learning while preserving data confidentiality through federated learning. Although still in their infancy, these models hold immense promise for advancing psychiatric research, particularly in the characterization and assessment of mood disorders. In this perspective paper, we articulate a forward-looking vision for deploying M3T-FedFMs in psychiatric practice and delineate key challenges and open research directions critical for realizing next-generation, AI-driven mental health care.

## 1 Introduction

Mood disorders (e.g., major depressive disorder and bipolar disorder) are among the most prevalent psychiatric conditions worldwide (1), affecting emotional and psychological well-being and contributing to adverse outcomes in occupational functioning, academic performance, and physical health (2). The profound and multifaceted impact of these disorders underscores the critical need for accurate diagnosis, effective treatment, and reliable prognosis. Traditionally, such processes have relied on clinical interviews conducted by psychiatrists, drawing heavily on patient self-reports and observational judgment. While this approach remains central to psychiatric practice, it is constrained by subjectivity, inter-clinician variability, and limited ability to capture temporal fluctuations in symptomatology (3).

To address these constraints, the research community has looked into the growing availability of digital health data – from electronic health records (EHRs) to wearable and smartphone-based monitoring – to enable the structured and longitudinal collection of both objective biomarkers and patient self-reports (4, 5). For example, EHRs store structured information such as diagnosis codes, lab results, medication histories, and standardized psychiatric assessments (e.g., Patient Health Questionnaire-9 (PHQ-9), Generalized Anxiety Disorder-7 (GAD-7)), while wearable devices and smartphones capture continuous streams of physiological and behavioral data, including heart rate, sleep patterns, physical activity, screen time, and even social interaction metrics (e.g., call and message frequency). Further, outside of the healthcare ecosystem, institutions such as schools and workplaces have begun collecting data that may signal the presence of mood disorders, including academic performance, attendance records, and productivity measures (6, 7). This unprecedented scale of data collection, coupled with advances in artificial intelligence (AI) and machine learning (ML), is enabling the integration of diverse data streams for the early detection, monitoring, and personalized treatment of mood disorders (8), marking a paradigm shift from traditional reliance on subjective self-reports toward data-driven, AI-assisted psychiatric care.

This paradigm shift is largely fueled by the remarkable ability of AI/ML algorithms to detect subtle and nonlinear relationships across clinical features, behavioral signals, and self-reported data, which are often difficult for human clinicians to discern (9). For instance, ML models have demonstrated the capacity to differentiate between major depressive and bipolar disorders (10), predict treatment responses (11), and forecast the trajectory of mood episodes by identifying early behavioral shifts that precede clinical deterioration (e.g., reduced location variability or disrupted sleep), thereby extracting clinically meaningful insights from noisy, real-world data (12). To provide a structured overview of the breadth of AI/ML integration in this domain, **Table 1** summarizes key AI/ML tasks explored for mood disorder care, organized across the domains of diagnosis, prognosis, treatment personalization, continuous monitoring, and clinical decision support. While these domains differ in nature from an AI/ML perspective (e.g., continuous monitoring vs. diagnostic classification), they overlap considerably from a psychiatric perspective and can be broadly aligned with the classical triad of diagnosis, prognosis, and treatment in clinical medicine.

**Table 1:** AI/ML tasks that support mood disorder analysis across diagnostic, prognostic, treatment, continuous monitoring, and clinical decision support domains.

Domain	AI/ML tasks for mood disorder care
Diagnostic Support	• Detecting presence of mood disorders • Assessing symptom severity (e.g., mild/moderate/severe depression) • Identifying comorbidities (e.g., substance use with mood disorders)
Prognosis & Risk Stratification	• Predicting relapse risk in mood disorders or recurrence of manic episodes • Forecasting illness trajectory (chronic vs. episodic course) • Estimating suicide/self-harm risk • Predicting polarity switches (depression → mania/hypomania in bipolar disorder)
Treatment Planning & Personalization	• Recommending optimal treatment (medication, psychotherapy, or combined) • Predicting individual response to specific antidepressants or mood stabilizers • Tailoring psychotherapy (e.g., CBT, interpersonal therapy) to patient profiles • Early identification of treatment-resistant cases
Continuous Monitoring & Early Detection	• Continuous mood tracking from speech, facial expression, or wearable data • Detecting early warning signs of relapse or manic onset • Monitoring sleep, circadian rhythms, and daily activity disruptions • Medication adherence and side-effect monitoring
Clinical Decision Support	• Providing real-time decision support for psychiatrists by summarizing or identifying data patterns • Assisting primary care physicians with screening and referral decisions • Risk predictions to support emergency/crisis triage • Monitoring treatment adherence and detecting protocol deviations
Research & Drug Development	• Identifying digital behavioral indicators (e.g., mobility patterns), biological measures, and neuroimaging markers (e.g., fMRI) relevant to mood disorders • Clinical trial optimization and patient matching • Cluster patients into clinically meaningful subtypes • Population-level treatment pattern analysis

### 1.1 Early signs of a new wave of innovation in AI/ML for mood disorders

The AI/ML field has witnessed a shift from conventional models (e.g., multilayer perceptrons and convolutional neural networks) toward more advanced model architectures, most notably large language models (LLMs) (13). In parallel, the psychiatric research community has begun exploring the use of LLMs for various applications, such as classifying depression severity from self-reports, social media posts, and clinical notes (14, 15). Beyond classification, LLMs have also been developed into conversational agents that provide emotional support and behavioral coaching (16), underscoring their capacity to assist in both diagnostic and therapeutic contexts. Although LLMs are often perceived as a recent innovation, they are already being superseded by another emerging class of models, called multimodal foundation models (FMs) (17), which represent one of the frontiers of the AI/ML field. In essence, the rise of multimodal FMs (e.g., GPT 5, Gemini 2.5 pro) was catalyzed by the limitations of conventional LLMs (e.g., GPT-3), which were inherently language-based models designed to process textual input and perform text-centric tasks (e.g., text summarization, generation, and correction). In contrast, multimodal FMs are designed to jointly process and integrate information from multiple data modalities (e.g., text, speech/audio, vision, and sensor data) and perform a diverse range of downstream tasks: for example, visual question answering and image captioning (i.e., vision–language tasks), emotion and speech recognition (i.e., audio–language tasks), and context-aware dialogue generation (i.e., cross-modal reasoning tasks). This integrative capacity is why they are often referred to as multimodal multitask foundation models (M3T-FMs) (17).

M3T-FMs are large-scale models pre-trained on vast and diverse datasets, granting them a broad contextual understanding of language, vision, audio, and other sensory modalities (17). This extensive pre-training enables the models to capture cross-modal correlations and generalizable representations that transfer effectively across tasks and domains. Once pre-trained, these models can be adapted to a wide range of downstream applications through various fine-tuning strategies, such as adapter-based tuning, low-rank adaptation (LoRA), and prompt-tuning (18), which allow efficient model customization without retraining the entire model. Although they remain relatively underexplored in psychiatry due to their recent emergence (19), M3T-FMs offer a new avenue for integrating heterogeneous/multimodal data streams that characterize mental health conditions. For instance, they can jointly model neuroimaging scans, unstructured clinician notes, social media activity, and therapy session recordings (including audio, video, and transcripts) to support diagnostic and therapeutic insights. Furthermore, they enable the fusion of multimodal data captured by smartphones and wearable devices (e.g., facial expressions, vocal tone, and inferred emotional states), capturing the features that are often disrupted in mood disorders. In addition, M3T-FMs can leverage data collected from the expanding Internet of Medical Things (IoMT) ecosystem (20), where connected sensors facilitate real-time physiological monitoring of indicators such as heart rate variability, sleep quality, and stress-related biomarkers, offering objective correlates of mood fluctuations.

Beyond their multimodal capabilities, M3T-FMs exhibit strong generalization and few-/zero-shot learning abilities (21), enabling them to seamlessly support the broad range of tasks summarized in **Table 1**. In particular, M3T-FMs can efficiently learn new tasks from limited examples (e.g., identifying emerging behavioral markers or interpreting novel linguistic cues in patient narratives) and rapidly adapt when the data encountered during deployment differ from those seen during training (i.e., under distribution shift). For example, a conventional model (e.g., a convolutional neural network) trained on pre-pandemic clinical data may fail to detect depression in patients during or after the COVID-19 pandemic, when emerging symptoms (e.g., health-related anxiety or prolonged isolation) became more salient predictors. In contrast, by providing only a few representative data samples of updated input (e.g., new therapy session transcripts or patient messages describing recent stressors), M3T-FMs can adjust their outputs without requiring full model retraining, a capability rarely attainable with conventional psychiatric AI systems.

### 1.2 Federated foundation models: unlocking the use of M3T-FMs in mood disorder analysis through federated learning

Although M3T-FMs hold significant promise for advancing mood disorder analysis in psychiatry, their effective deployment requires careful consideration of implementation nuances. In particular, to fully realize their potential in this domain, M3T-FMs must be fine-tuned on domain-specific data (or, in some cases, trained from scratch using psychiatric datasets). However, the conventional training pipelines of these models are inherently centralized (i.e., fine-tuning requires data from multiple institutions to be gathered in a central server). For instance, geographically-distributed psychiatric clinics and IoMT devices would need to transfer sensitive patient data to a shared cloud infrastructure for model training. Such centralization is often impractical and ethically problematic, as psychiatric data contain deeply personal information related to mental health symptoms, treatment history, and behavioral patterns. Consequently, such data transfers may violate privacy regulations (e.g., the Health Insurance Portability and Accountability Act (HIPAA) (22), the General Data Protection Regulation (GDPR) (23)) and undermine patient trust. As a result, the very nature of M3T-FM training presents a fundamental barrier to their clinical adoption for mood disorder analysis, raising a critical research question: how can we enable the collaborative training and adaptation of M3T-FMs across various data-collecting entities without directly sharing sensitive psychiatric data?

The key to addressing the above question lies in integrating distributed learning methods into the training pipeline of M3T-FMs. Among such methods, federated learning (FL) offers a particularly promising solution. FL enables AI/ML models to be trained across decentralized data sources (e.g., hospitals, clinics, and personal devices) without requiring the raw data to be transferred to a central server. In an FL setting, each participating entity (or client) trains the model locally on its own data and transmits only model updates (e.g., gradients or weights) to a central coordinator. The server then aggregates these updates (e.g., through weighted averaging of parameters) and distributes the updated global model back to all entities. Each entity subsequently synchronizes its local model with the global model and initiates the next round of local training. This iterative cycle continues until the global model converges. In particular, FL avoids the transfer of raw data across the network since only model updates (instead of the underlying data) are exchanged among participating entities. This makes combining FL with M3T-FMs particularly appealing and has recently marked the emergence of a new paradigm in AI/ML, known as federated foundation models (FedFMs) (24, 25), or more specifically, multimodal multitask federated foundation models (M3T-FedFMs) (26–29). M3T-FedFMs remain entirely unexplored in psychiatry and mood disorder analysis, forming the cornerstone of the motivation behind this work.

It is worth noting that FL, in its native form, was originally designed for conventional deep learning architectures (e.g., convolutional neural networks and multilayer perceptrons) and typically applied to single-modality data. Following this native form, in psychiatry, FL has been used for tasks such as mood disorder detection (30, 31), risk identification (32), and treatment support (33). Nevertheless, the integration of FL with M3T-FMs, forming M3T-FedFMs, remains unexplored in psychiatry and mood disorder research. In this work, we aim to (i) introduce M3T-FedFMs to the research community in psychiatry and mood disorders, (ii) shed light on their tremendous potential for advancing this domain, and (iii) identify key challenges and open research directions that must be addressed to fully realize their impact.

In the following, given the lack of prior work on the application of M3T-FedFMs in psychiatry and the mood disorder community, we first introduce a modular architecture for M3T-FedFMs and outline their deployment design.

## 2 M3T-FedFMs architecture and deployment design for mood disorder care

**Figure 1:**
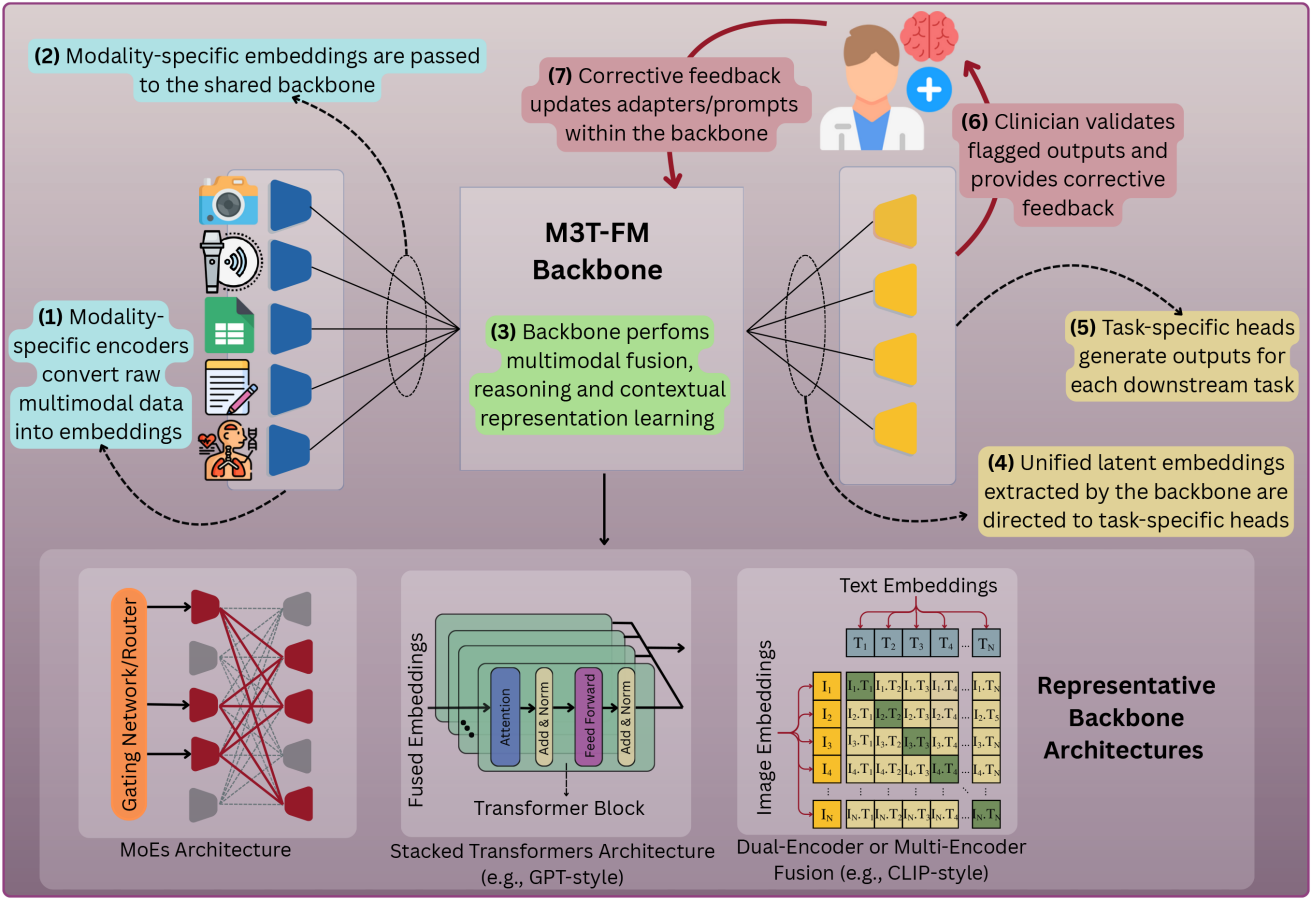
Schematic of an M3T-FM architecture visualizing the end-to-end workflow. The text within the figure explains the data flow and the HITL design in M3T-FMs. Light-cyan boxes (Steps 1–2) represent input encoding, the light-green box (Step 3) denotes backbone processing, light-yellow boxes (Steps 4–5) indicate task-specific output generation, and light-red boxes (Steps 6–7) illustrate the Human-in-the-Loop (HITL) feedback mechanism for continuous model refinement.

In a nutshell, M3T-FedFMs entail the distributed fine-tuning of M3T-FMs across a set of clients or data-collecting entities. Therefore, understanding M3T-FedFMs first requires an understanding of the underlying architecture of M3T-FMs. Nevertheless, due to their emerging nature, M3T-FMs currently lack a unified architectural standard and remain an area of active development. To provide a generalizable perspective, we present a modular architecture for M3T-FMs, composed of various key components (illustrated in **Figure 1**), inspired by recent advances in foundation model design (34). This modular design enables independent updates and adaptations of the model’s constituent components. In what follows, we first describe these five components in detail, then explain how M3T-FedFMs perform distributed training across clients, and finally discuss a critical requirement for deploying M3T-FedFMs in psychiatry and mood disorder analysis.

### 2.1 Key components of an M3T-FM and model fine-tuning techniques

The key components of an M3T-FM are as follows:

1. Modality Encoders: M3T-FMs employ dedicated modality encoders that convert raw inputs (e.g., speech waveforms, facial images, or text) into standardized intermediate embeddings. The generated embeddings are then integrated and passed to the backbone of the model for joint representation learning. This design allows clients to activate or fine-tune only the encoders relevant to the modalities they have available, rather than relying on a fixed set of input types.

2. Shared Backbone: The shared backbone acts as the main part of the model that receives the modality specific embeddings and performs multimodal fusion, reasoning, and contextual representation learning. This is central to a model’s ability to generalize across different clients and tasks, and it can take several architectural forms depending on the model’s design goals, three of which are described below:

- Stacked Transformers Architecture (e.g., GPT-style (35)): This architecture uses a stack of transformers to process all incoming data embeddings together. It is highly effective for tightly integrating information and is well-suited for large-scale pretraining on extensive, multimodal datasets. However, this approach can be computationally demanding, which is important in psychiatry as related institutions such as psychiatric clinics may lack powerful/extensive computational resources (e.g., small rural clinics).
- Dual-Encoder or Multi-Encoder Fusion Architecture (e.g., CLIP-style (36)*)*: In this design, each data modality is processed by its own dedicated encoder/backbone stream. The representations formed by these separate encoders are then aligned and combined in a shared latent space, often through techniques such as contrastive learning or cross-attention (37). This modular approach is well-suited for clinical psychiatry, as it enables flexible model adaptation in cases where certain data streams or modalities are missing in some clients (e.g., a clinic may lack video-based facial data but possess audio recordings or therapy-session transcripts, which can still be processed and fused through dedicated encoders and multimodal fusion mechanisms).
- Mixture-of-Experts (MoEs) Architecture (34): This architecture employs a collection of specialized “expert” sub-networks that are selectively activated based on the client’s data (e.g., patient’s clinical records), training context (e.g., clinical context), or task requirements (e.g., mood disorder diagnosis). A set of gating networks dynamically routes the fused representations through the most relevant expert pathways within the backbone. This design is particularly advantageous for psychiatric institutions that often operate with limited infrastructural and computational resources, as it enables the model to remain efficient by activating only a small subset of expert components tailored to each patient’s profile.

3. Task Heads: M3T-FMs include dedicated task heads that transform the representations produced by the backbone into task-specific outputs (e.g., classification of depression severity, prediction of treatment response, or estimation of relapse risk). The modular design of these task heads enables targeted model adaptation while keeping the backbone fixed, allowing efficient fine-tuning for new tasks or populations. Moreover, these heads can be easily swapped or personalized across clients without retraining the entire model.

Due to the large size of M3T-FMs (e.g., models containing trillions of parameters), it is often infeasible to train their aforementioned components through full model updates. Thus, to enable personalization at clients without retraining the entire backbone, M3T-FMs incorporate lightweight adaptation strategies, commonly referred to as parameter-efficient fine-tuning (PEFT) techniques. Three complementary PEFT approaches are particularly well-suited for psychiatric applications, described below:

- Adapter-based Fine-Tuning (38): Adapters are compact *trainable* modules inserted between layers of the frozen backbone. This approach retains the backbone’s pre-trained knowledge while significantly reducing computational costs. In psychiatric applications, adapters make it possible for each institution or device to fine-tune the model’s behavior locally while keeping most of the backbone frozen/fixed. This substantially reduces computational overhead during model training.
- LoRA (Low-Rank Adaptation) (39): This technique decomposes model parameter-update matrices into low-rank factors, allowing fine-tuning through a minimal number of trainable parameters. LoRA is shown to nearly maintain the expressiveness of full model fine-tuning (39) while significantly reducing memory footprint and training cost, making it practical for clinics or edge devices with constrained resources.
- Prompt-Tuning (40): Rather than modifying internal parameters of the model, prompt-tuning introduces learnable input tokens into the sequence of modality embeddings or inserts them at strategic locations inside the model. These tokens act as instructions guiding the model toward task- or patient-specific behavior. In mood-disorder care, prompt-tuning allows personalization of outputs (e.g., severity classification or treatment recommendations) using local patient data without altering the foundation model’s core parameters.

Research on the above three PEFT techniques remains highly active (41), as each presents distinct trade-offs in memory efficiency, computational cost, and representational flexibility. Consequently, all of them are feasible choices for deployment, with their relative advantages depending on the specific application context and the continually evolving state of the art.

### 2.2 Learning coordination paradigm of M3T-FedFMs

The above components define the architecture of an M3T-FM, which serves as the building block for M3T-FedFMs. Specifically, in a psychiatric ecosystem powered by M3T-FedFMs, each participating client (e.g., psychiatric clinics, hospitals, or wearable devices) retains its own data and performs local training on its M3T-FM using the aforementioned fine-tuning techniques. Following local model training, clients engage in a coordination process to enhance their model generalization: each client transmits its locally updated parameters (e.g., adapters or prompts) to a central server (e.g., a cloud server), which aggregates the updates (typically via weighted averaging of parameters) and then distributes the resulting global model back to all clients. This iterative process continues until the global model converges.

In practice, the nature/type of participating clients can vary substantially in M3T-FedFMs. In turn, one of the key advantages of M3T-FedFMs lies in their flexibility for deployment across diverse client types (whether individual devices, clinical institutions, or hybrid combinations of both), each carrying distinct implications for psychiatric care and data governance:

- Cross-Device M3T-FedFMs: In this setting, a large number of IoMT devices (e.g., smartphones, sensors, wearables) participate in training a shared model while keeping their data local (42). This paradigm involves massively distributed and heterogeneous devices with limited computational resources and intermittent connectivity. In psychiatric care, cross-device M3T-FedFMs are well-suited for integrating patient-generated data streams such as sleep patterns, speech features, or behavioral signals from personal IoMT devices.
- Cross-Silo M3T-FedFMs: In cross-silo M3T-FedFMs, participating entities are organizations or institutions (e.g., hospitals, clinics, schools), each holding large volumes of data (43). The number of clients is smaller here, but more stable and reliable than in cross-device settings. Cross-silo M3T-FedFMs is particularly valuable for combining institutional datasets, such as EHRs or structured clinical assessments in different clinics, while maintaining privacy and compliance with regulations.
- Hybrid M3T-FedFMs: This setting blends the above two approaches, involving multiple silos (e.g., hospitals) as well as many edge devices (e.g., wearable devices of patients). Here, coordination and learning occur at multiple levels, often requiring hierarchical aggregation strategies and specialized privacy mechanisms (44). In psychiatric care, hybrid M3T-FedFMs are especially powerful because they enable the integration of institutional datasets (e.g., from clinics or hospitals) with real-world signals captured from patients’ daily lives through personal IoMT devices such as wearables and smartphones.

### 2.3 M3T-FedFM deployment through human-in-the-loop oversight and feedback

In addition to the technical components described above, safe deployment of M3T-FedFMs in psychiatry requires embedding a human-in-the-loop (HITL) mechanism as a fundamental part of the system design. In this design, qualified experts (e.g., psychiatrists) systematically review model outputs, validate or reject them, and provide corrective feedback that is incorporated into lightweight components of the model (e.g., adapters, prompts, or task heads). Embedding this oversight ensures that M3T-FedFMs remain clinically reliable while continually improving through expert-guided adaptation.

HITL implementation can vary depending on the deployment context and the specific use cases for which M3T-FedFMs are applied. While the next section provides a discussion of these contexts, here we illustrate two representative scenarios that demonstrate how HITL mechanisms can be integrated into practice:

- Institutional Deployment: When M3T-FedFMs are deployed within organizations (e.g., healthcare or educational institutions), in-house experts review alerts and predictions produced by the local M3T-FMs. For example, if a model flags an individual as being at-risk based on a behavioral or linguistic pattern, a clinician or expert evaluates the evidence and decides whether the assessment is clinically meaningful. The feedback of the expert is then used to refine the model, aligning its predictions more closely with clinical standards.
- Personal/Consumer Deployment: When M3T-FedFMs are deployed directly on personal devices (e.g., smartphones or wearables), HITL oversight requires secure transmission of selected inputs and outputs of the local M3T-FMs to trusted experts for review. For instance, an M3T-FM-powered app may track mood using questionnaires, speech tone, and physiological signals, but flagged high-risk cases (e.g., elevated suicide risk) are automatically routed to a psychiatrist for validation, ensuring that high-risk predictions and resulting clinical decisions remain under human authority.

By embedding HITL mechanisms, M3T-FedFMs can safely operate as decision-support systems (rather than as autonomous agents) for mood disorder analysis. This design preserves essential human oversight in safety-critical psychiatric applications while allowing the models to continuously benefit from expert feedback. Having outlined the overall framework, we next turn to real-world use cases that illustrate how M3T-FedFMs can be applied to enhance psychiatric support for mood disorders.

## 3 Potential use cases of M3T-FedFMs in mood disorder care

Through the following use cases, we aim to demonstrate how M3T-FedFMs can be deployed to enhance psychiatric care for mood disorders across multiple domains. To this end, we first outline a set of forward-looking use cases of M3T-FMs and then describe how their FL-driven fine-tuning, envisioned in M3T-FedFMs, further strengthens and operationalizes each use case in practice. **Table 2** provides an overview of these scenarios, illustrating the wide range of data modalities collected across them and the corresponding psychiatric applications and tasks.

**Table 2:** Representative environments/settings aligned with the proposed use-cases of M3T-FedFMs in mood-disorder psychiatry.

Setting	Established modalities	Emerging modalities	Representative psychiatric applications
Psychiatric Clinics (45–47)	Text (EHR, clinician notes), structured/tabular (scales, demographics), physiological (heart-rate during therapy)	Audio (speech-derived cues), visual (facial and gestural cues), neurophysiological (neurofeedback signals during cognitive-behavioral therapy), eye-tracking (gaze and pupil-dynamics), motion and haptic (body-movement and touch-based affect signals)	Diagnosis support; longitudinal symptom tracking; treatment personalization
Hospitals (48–50)	Neuroimaging (MRI, fMRI), electrophysiological (EEG, ECG), structured/tabular (lab reports, demographics), text (clinical reports), physiological (vital signs)	Functional near-infrared spectroscopy (fNIRS), voice (speech and prosody), visual (behavioral monitoring during inpatient sessions), digital cognitive-performance assessments (reaction-time and attention-task data)	Brain-behavior modeling (48); comorbidity prognosis; identification of neuroimaging and physiological markers
Schools/ Universities (51)	Text (survey responses, teacher assessments), structured/tabular (grades, attendance)	Audio (classroom or counseling interactions), visual (classroom engagement or counseling video recordings), physiological (stress and arousal), eye-tracking (attention and fatigue), digital-behavioral data from learning platforms	Early detection of at-risk students; linking academic performance and well-being
Workplaces (7, 52, 53)	Text (employee surveys, occupational health records), structured/tabular (productivity, absenteeism)	Audio (vocal features during meetings), visual (facial expressions from virtual meetings), behavioral-kinematic (typing, mouse, and interaction dynamics), physiological (heart rate, sleep), digital collaboration patterns (messaging frequency, response times)	Screening for burnout and stress; evaluation of workplace well-being programs
Personal Devices (4, 12)	Physiological (sleep, activity level), motion (accelerometer and gyroscope), digital-behavioral (app usage, GPS, screen time)	Visual (front-camera affect and micro-expression), voice (prosody, speech biomarkers), contextual or environmental (ambient light, sound, IoT context)	Passive mood monitoring; early relapse detection; adaptive intervention timing
AI Companions (54, 55)	Text (dialogues), audio (prosody, tone), visual (facial expression, gaze, gesture)	Physiological (wearable-linked bio-signals), environmental context (ambient sound, lighting, location), haptic feedback (touch-based interaction for embodied agents)	Conversational therapy; daily mood tracking; social-skill rehearsal

Each setting is characterized by its established and emerging modalities, psychiatric relevance, and maturity of digital deployment.

*Established modalities* denote data types that are mature and commonly integrated within psychiatric or research workflows. *Emerging modalities* represent under-integrated but increasingly informative data sources expanding the multimodal potential of M3T-FedFMs. It is worth noting that several emerging modalities, such as audio, video, and other high-sensitivity data streams, are feasible only when collected under explicit consent procedures and ethical or institutional approval mechanisms, and such frameworks are expected to become more developed and standardized in the future.

(Use Case 1) Next-Generation Psychiatric Clinics: M3T-FMs could enable intelligent assistants in psychiatric clinics that synthesize multimodal patient data to support mood disorder analysis. During routine sessions, these assistants could process diverse inputs such as speech, facial expressions, verbal content (e.g., responses to standardized psychiatric questionnaires), and physiological biomarkers collected through wearable devices. By fusing these multimodal data sources, the M3T-FM can construct a real-time, fine-grained representation of the patient’s mental state. This enables the system to detect subtle behavioral shifts, mood fluctuations, or early warning signs of relapse that might otherwise go unnoticed. These assistants can support critical clinical tasks such as identifying early depressive episodes, flagging elevated suicide risk, or estimating treatment response (see **Table 1**). Note that these models are *not* claimed to replace clinicians but rather to augment clinical decision-making by highlighting patterns and longitudinal trends that are difficult for humans to detect unaided.

(Use Case 2) Bridging Physical and Mental Health in Hospitals: Physical and mental health are strongly interconnected (56) and this relationship is bidirectional: on the one hand, mood disorders can negatively affect physical health by worsening sleep quality, impacting blood pressure, or weakening immune responses (57). On the other hand, physical illnesses can significantly influence a patient’s mood. For instance, individuals diagnosed with conditions such as cancer or cardiovascular disease often experience hopelessness or loss of motivation, which can lead to depression as a secondary consequence of their illness (58). Implementing M3T-FMs in hospitals thus offers a unique opportunity to gain insights from this bidirectional relationship. Specifically, by integrating multimodal medical data such as biomedical biomarkers (e.g., blood pressure, heart rate), EHRs, neuroimaging results, and patients’ medical histories, psychiatric systems enabled by M3T-FMs could support mood disorder analysis inside the hospitals. For example, an M3T-FMs-powered system might detect that a patient undergoing chemotherapy is experiencing declining sleep quality and reduced activity levels, which are patterns associated with elevated risk of depression. The model could then flag this risk to the clinical care team, prompting timely psychological evaluation and intervention alongside physical treatment.

(Use Case 3) School-Based Screening and Support: Mood disorders often begin to manifest during adolescence or early adulthood, even though formal diagnosis typically occurs later in life (59), making early screening in schools and universities critically important. Further, research shows that many mood disorders, such as bipolar disorder and major depression, sometimes have genetic and neurodevelopmental components (60), which are often hard to detect. For example, a student may carry a genetic predisposition yet present only subtle or atypical symptoms, making detection difficult without structured observations. Compounding this challenge, children and adolescents often have limited emotional awareness or vocabulary to articulate their internal experiences, resulting in undetected distress (61). Subsequently, an M3T-FM-powered system deployed in educational settings (e.g., schools, universities) could support early screening and timely intervention of mood disorders. These systems could integrate multimodal student data such as classroom behavior observations, speech or writing patterns, attendance trends, and academic performance metrics (e.g., GPA or sudden grade fluctuations). By fusing these modalities, the model may uncover patterns consistent with emerging mood disorders. For example, it might detect that a student’s classroom participation has dropped significantly, their recent writing includes negative or hopeless language, and their academic performance has declined – together indicating potential risk. The system could then flag the student for follow-up by school counselors or psychiatrists, enabling earlier support and reducing long-term impact.

(Use Case 4) Workplace Mood Monitoring: Mood disorders can emerge or worsen in environments characterized by chronic stress and high workload (52). Specifically, research has consistently shown that prolonged exposure to occupational stressors significantly increases the risk of depression and burnout (62). The healthcare sector provides a clear example of such a high-stress environment: physicians, nurses, and psychiatrists frequently experience emotional exhaustion, long hours, and intense pressure – factors that heighten their vulnerability to mood disorders (63). Subsequently, implementing M3T-FMs in these settings offers a promising avenue for proactive mental health support. These models could function as *intelligent workplace assistants* that monitor mental health indicators and detect early signs of deterioration. For instance, M3T-FMs could analyze data such as periodic self-assessments, vocal features during meetings or check-ins, and patterns in written communication (e.g., emails). By integrating these signals, the model could estimate an employee’s mood state and flag early indicators of depression, burnout, or emotional fatigue. The system could then prompt adaptive interventions, such as recommending rest, offering personalized coping strategies, or alerting occupational health teams based on these insights.

(Use Case 5) Everyday Mental Health Agents: In psychiatry, patients spend most of their time outside clinical settings, where daily routines unfold and symptoms such as mood swings or fatigue often emerge without clinician oversight (64). Therefore, everyday personal environments represent vital contexts for psychiatric care and offer important opportunities for monitoring and supporting mental health (65). To address this need, M3T-FedFMs can enable the development of intelligent, supportive systems that *assist patients with mood disorders throughout treatment* in these settings. For instance, an application powered by M3T-FedFMs running on smartphones or wearables could passively collect behavioral signals such as sleep patterns, activity levels, or changes in typing speed and communication patterns. Such systems may also incorporate patient self-reports (e.g., brief mood check-ins) as well as gestures and facial expressions captured during video sessions. By integrating these inputs, M3T-FedFMs can monitor emotional states over time, predict treatment response, and deliver personalized recommendations: for example, evidence-based suggestions such as mindfulness exercises during stressful periods or prompts to contact a clinician when early signs of relapse appear. In urgent cases, the system may flag concerning patterns for clinician review through established, consent-based protocols, ensuring that any intervention remains under human oversight while extending psychiatric support beyond the clinic and into everyday life.

(Use Case 6) AI Companions: AI companions represent a new horizon for psychiatric care (66), offering supportive, personalized, and continuous engagement. When powered by M3T-FedFMs, these systems have strong potential to transform care across both clinical and home environments. Unlike conventional mobile health applications, which rely on reactive user input (67), AI companions can create immersive, adaptive, and sustained interventions that are especially relevant for mood disorders. Two emerging directions illustrate this potential:

- Extended Reality (XR): XR applications powered by M3T-FMs, recently recognized as a feasible paradigm in AI/ML field (27), can be delivered through virtual reality (VR), augmented reality (AR), and mixed reality (MR) devices to immerse patients in therapeutic environments tailored to their emotional states while preserving privacy. For example, a patient with bipolar disorder in a depressive episode may avoid in-person sessions due to hopelessness or fatigue. An XR system could instead create a calming and socially supportive virtual setting. By integrating multimodal signals, such as speech tone, gaze, and physiological markers from wearables, the model can adapt the experience in real-time, offering relaxation exercises, graded social exposure, or cognitive interventions aligned with the patient’s condition.
- Embodied AI: Embodied AI agents powered by M3T-FMs represent another recently recognized and feasible paradigm in the AI/ML field (26). When adapted to psychiatry, robots, avatars, or screen-based agents powered by M3T-FMs can interact with patients through speech, facial expressions, and gestures, creating a more natural and engaging experience. These companions can detect emotional cues in real-time and respond empathetically. For example, they may remind patients to maintain regular routines (such as consistent sleep and meals), suggest coping strategies for stress or negative thoughts, prompt medication adherence, or escalate urgent concerns to clinical teams when necessary. Moreover, these companions can anticipate potential relapses and proactively initiate supportive actions, such as suggesting therapy check-ins, recommending behavioral interventions, or prompting patients to contact their clinicians. As a result, by embedding psychiatric support into everyday interactions, embodied AI agents can bridge the gap between clinical encounters and daily life, shifting AI companions from passive helpers to active partners in long-term psychiatric care.

### 3.1 Enabling the aforementioned use cases through M3T-FedFMs

Together, the aforementioned applications highlight the broad potential of M3T-FedFMs to transform mood disorder care across clinical, educational, occupational, and home domains. However, realizing this potential faces a fundamental challenge: mood-related data is inherently distributed across diverse clients (e.g., institutions and personal devices), each holding unique but incomplete views of an individual’s mental health state. As a result, training an M3T-FM locally within a single client is of limited practicality, as models trained on small or biased datasets may lack generalizability. For instance, a model trained solely on data from a single urban psychiatric clinic may fail to generalize to a new patient from a rural population with different socioeconomic backgrounds, cultural contexts, or comorbidity patterns. Similarly, a mobile device that captures only a single data modality, such as activity levels or voice tone, may provide limited insight on its own, yet when combined with complementary data collected by other clients (e.g., clinical notes, wearable signals, or therapy transcripts), it can contribute to a more holistic and temporally continuous understanding of the patient’s mood dynamics. Combining data from multiple clients could address these limitations by providing broader population representation and richer modality coverage. However, this requires aggregating sensitive psychiatric data on shared servers, creating significant privacy risks and potential violations of regulations such as HIPAA and GDPR. Therefore, across all the use cases outlined above, M3T-FMs must be paired with FL-driven fine-tuning, forming M3T-FedFMs, to ensure that these models remain both effective and ethically deployable in real-world psychiatric settings.

**Figure 2:**
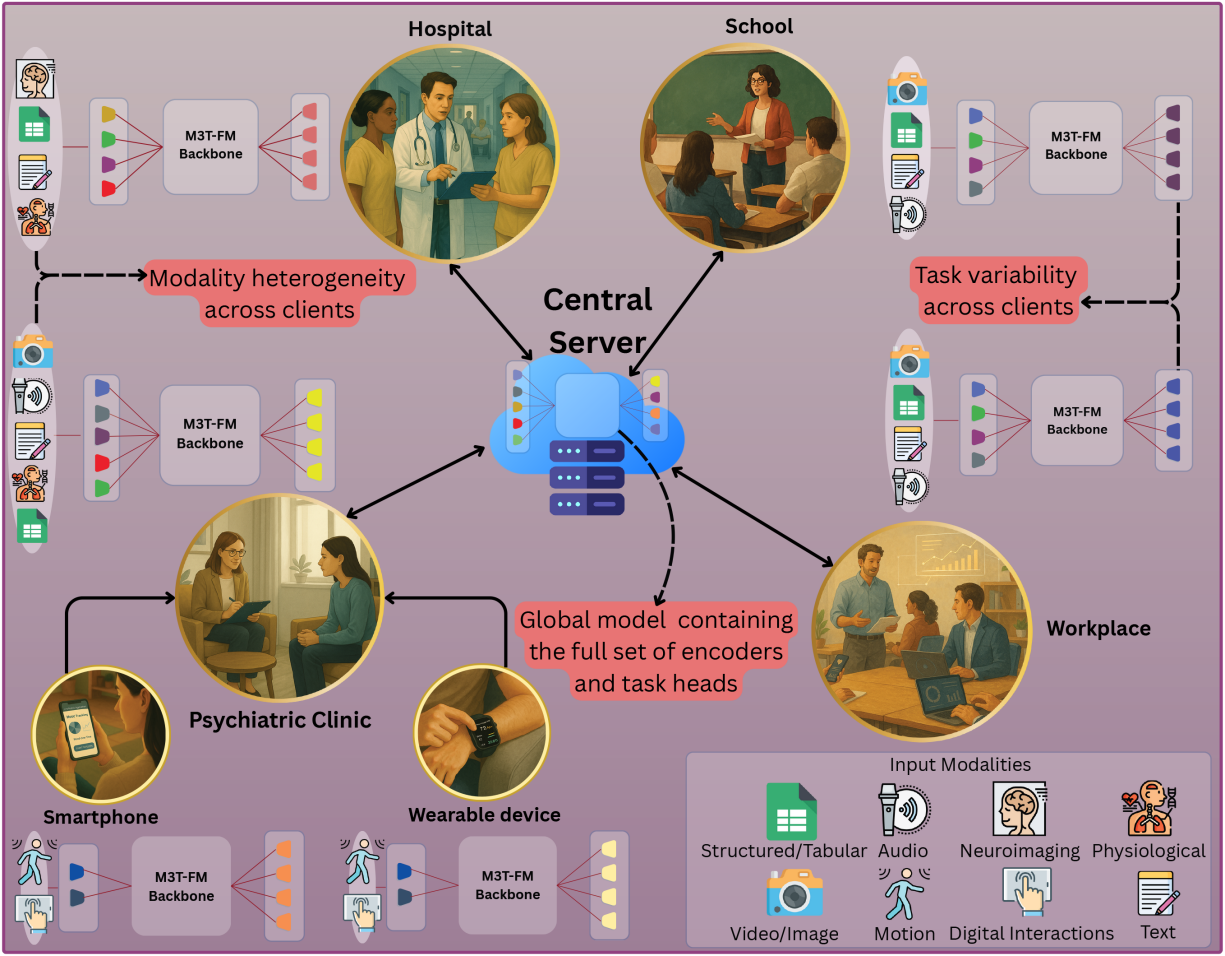
A schematic of M3T-FedFMs deployment. Personal devices (e.g., smartphones, wearables) first train local models on patient data, which are aggregated by their respective institutional servers (e.g., psychiatric clinics). These silos then collaborate with other institutions from different domains (psychiatric clinics, hospitals, schools, workplaces), each training on its own multimodal data. Finally, a central aggregator combines updates from both devices and silos to form a global M3T-FedFM that integrates cross-sector knowledge while preserving privacy.

In particular, in the first use case mentioned above, M3T-FedFMs unlock access to broader and more diverse patient populations across psychiatric clinics, allowing intelligent clinical assistants to learn from multimodal behavioral and physiological data distributed across sites without breaching data privacy. In the second use case, deploying M3T-FedFMs across hospitals enables joint learning from interconnected physical and mental health data, such as biomedical signals, neuroimaging, and EHRs, thereby improving the detection of mood changes associated with comorbid medical conditions. In the third use case, M3T-FedFMs facilitate privacy-preserving collaboration among schools and universities, allowing models to detect early signs of mood disorders while ensuring that sensitive student information never leaves institutional boundaries. In the fourth use case, M3T-FedFMs enable decentralized learning across organizations, capturing shared patterns of stress, burnout, and emotional fatigue without exposing personal communication data or violating corporate confidentiality. In the fifth use case, M3T-FedFMs allow smartphones, wearables, and personal devices to collaboratively learn from continuous behavioral and emotional data, improving the accuracy of mood predictions while keeping user data local and secure. Finally, in the sixth use case, M3T-FedFMs empower AI companions to evolve collectively across users and environments, learning generalizable behavioral patterns while maintaining on-device model personalization essential for safety-critical psychiatric care. To ease understanding, in **Figure 2**, we illustrate how M3T-FedFMs integrate data from multiple domains to provide a comprehensive view of patients’ mood and mental health state.

## 4 Technical and ethical challenges in deploying M3T-FedFMs for mood disorder care

Despite their tremendous potential, deploying M3T-FedFMs in psychiatry poses a range of ethical and technical challenges as AI/ML models in this domain must minimize diagnostic errors, act reliably when interacting with patients, preserve patient privacy, handle heterogeneous data modalities and diverse tasks, and simultaneously enable personalization through continuous adaptation to local contexts. As a result, in the following, we outline several major categories of challenges and corresponding research directions for deploying M3T-FedFMs in psychiatry for mood disorder care.

### 4.1 (Challenge 1) limits of human-in-the-loop design: protocols and accountability

Although HITL oversight is essential for ensuring the safe deployment of M3T-FedFMs in psychiatry, it faces practical challenges related to both scalability and responsibility. In particular, clinicians cannot feasibly review every model’s output given the sheer volume of multimodal data and limited psychiatric resources. To address this, AI/ML systems should rely on risk stratification protocols (68), where only a subset of outputs – such as high-risk cases, low-confidence predictions, or those tied to specific clinical indicators – are escalated for expert review. While this approach improves efficiency, it introduces a critical trade-off: if thresholds are too strict, important warning signs may be overlooked; if too loose, clinicians face alert fatigue and oversight burden, undermining the very safety that HITL is designed to provide. Future research must therefore develop precise and evidence-based stratification protocols (69) for M3T-FedFMs to minimize these risks. Here, it is important to note that, even with well-designed protocols, oversight gaps are unavoidable (e.g., not every data stream can be continuously monitored), which raises difficult questions of liability. Specifically, when adverse outcomes occur – whether because a critical case was never escalated to a clinician (e.g., a patient at high risk of suicide misclassified as low risk), or because an incorrect model output was accepted without review (e.g., a model recommending discontinuation of treatment that a clinician would have rejected) – responsibility becomes ambiguous. In these settings, it remains unclear whether responsibility rests with the supervising clinician, the deploying institution, or the model developers. This ambiguity underscores the urgent need not only for improved risk stratification protocols but also for clear regulatory and legal frameworks that define accountability and safeguard patients in psychiatric applications of M3T-FedFMs.

### 4.2 (Challenge 2) explainability and transparency

In psychiatry, model explainability is a core requirement for the clinical adoption and responsible use of AI models. In particular, psychiatrists must be able to understand why a model produces a specific prediction – such as flagging a patient as high risk for depressive relapse or recommending a particular intervention – in order to confidently integrate that output into their clinical decision-making. Nevertheless, M3T-FedFMs are typically built on massive-scale neural network architectures with billions/trillions of parameters. While these models can capture highly complex patterns across speech, language, physiological data, and behavior, their power comes at the cost of transparency: their internal decision-making processes are often opaque, earning them the reputation of ‘‘black box” systems (70).

It is worth noting that explainable AI (XAI) methods aim to make AI/ML model predictions more transparent (71, 72). For example, techniques such as SHapley Additive exPlanations (SHAP) (73) estimate the relative contribution of input features to a model’s prediction. However, most existing XAI methods are designed for single-modality data and conventional AI/ML architectures, and thus fall short when applied to complex models such as M3T-FedFMs. Specifically, the multimodal nature of M3T-FedFMs requires XAI techniques that can integrate heterogeneous data streams, such as speech, physiological signals, behavioral metrics, and self-reports, each operating on different temporal and semantic scales. This is despite the fact that standard XAI methods such as SHAP can identify important features within each modality, but in psychiatry, clinicians require more than isolated feature importance lists from M3T-FedFMs; they seek a narrative that explains how multiple signals interact and evolve over time to justify a diagnosis or alert. For instance, a suicidality risk prediction made by M3T-FedFMs becomes meaningful only if the model can illustrate how increased use of hopeless language in therapy transcripts, reduced social engagement inferred from mobile sensing data, and disrupted sleep patterns jointly indicate clinical deterioration over time. A key research direction, therefore, lies in developing multimodal temporal XAI frameworks for M3T-FedFMs that can capture cross-modal interactions, causal dependencies, and temporal dynamics of mood-related signals, transforming model outputs into clinically transparent narratives that align with psychiatric reasoning and support trustworthy decision-making.

### 4.3 (Challenge 3) algorithmic bias and cross-population fairness

Although M3T-FedFMs can improve model generalizability by leveraging geographically distributed data, they remain vulnerable to systematic bias arising from multiple sources. First, the pre-training of M3T-FMs is typically conducted on large-scale internet-derived datasets, which may underrepresent or misrepresent certain demographic groups with specific cultural backgrounds. For example, as mental health expressions vary significantly across cultures, if pre-training corpora predominantly reflect Western cultural norms and linguistic expressions of mental health, models may fail to recognize culturally specific manifestations of mood disorders in other populations (74). Second, bias may emerge from data imbalance across participating clients in the FL environment, affecting the fine-tuning process of M3T-FedFMs. Specifically, if the contributing institutions primarily serve specific demographic groups (e.g., urban populations, high-income individuals, or particular age cohorts), the aggregated global model may become skewed toward these dominant groups. For instance, an M3T-FedFM developed primarily using data from urban psychiatric clinics may generalize poorly to rural clinics, where patient demographics and socioeconomic contexts differ substantially. Addressing these sources of bias requires the development of fairness-aware learning mechanisms within M3T-FedFMs. Promising research directions include fairness-aware model aggregation and weighting strategies for M3T-FedFMs that explicitly balance contributions across demographic subgroups, preventing dominant populations from overshadowing minority groups (75). In addition, domain-adaptive and cross-lingual transfer learning techniques can be explored to improve generalization across languages, cultural contexts, and region-specific expressions of mood disorders (76). Integrating such approaches into M3T-FedFMs is essential for ensuring equitable and clinically valid deployment across diverse populations.

### 4.4 (Challenge 4) security and adversarial robustness

While M3T-FedFMs mitigate privacy risks by retaining raw psychiatric data at local institutions, they remain vulnerable to several security and confidentiality threats inherent to FL environments. A primary concern in this context is the reliability and integrity of client-side data and model updates, where a common adversarial threats arising from this issue is model poisoning (77). In such attacks, malicious participants deliberately manipulate their local training data or gradient updates before transmission to the central server, thereby corrupting the model aggregation process, implanting backdoors, or degrading overall predictive performance. To mitigate poisoning attacks, robust model aggregation mechanisms, such as Byzantine-resilient methods (e.g., trimmed mean and Krum), can be employed to reduce the influence of anomalous or malicious updates (78). However, in psychiatric applications, distinguishing client adversarial behavior from legitimate data heterogeneity across clients remains challenging, as patient populations often exhibit substantial clinical and demographic variability.

In addition to active manipulation, FL systems are also susceptible to information leakage during the model exchange/aggregation process: although raw data are not shared, transmitted model updates may unintentionally reveal sensitive patient information. In particular, prior work has shown that adversaries can exploit the exchanged model updates through gradient inversion attacks to reconstruct features of patients’ records (79) or through membership inference attacks to determine individuals’ participation in training (80). Such vulnerabilities raise notable concerns regarding patient confidentiality and regulatory compliance. To mitigate these risks, complementary defense mechanisms must be integrated into M3T-FedFMs. For example, secure multi-party computation (SMPC) provides a layer of protection by enabling encrypted aggregation of model updates through cryptographic protocols such as secret sharing and homomorphic encryption (81, 82). Nevertheless, while SMPC strengthens confidentiality, it introduces substantial computational and communication overhead, limiting its feasibility in resource-constrained psychiatric environments. Further, differential privacy (DP) provides an additional layer of protection by reducing the likelihood of reconstruction and inference attacks by injecting calibrated noise into transmitted model updates (83). However, stronger privacy guarantees often require higher noise levels, which may impair model accuracy and convergence. This is despite the fact that even modest performance degradation can compromise early risk detection and decision-making in clinical settings. In conclusion, future research can focus on developing integrated solutions tailored to the deployment of M3T-FedFMs in psychiatric environments, including (i) authentication and integrity verification of client-side model updates, (ii) robust model aggregation strategies to prevent compromised sites/clients from disproportionately influencing the global model, (iii) adaptive privacy mechanisms that balance noise and accuracy for M3T-FedFMs, and (iv) lightweight cryptographic protocols optimized for large-scale models encountered in M3T-FedFM setups. Across all of these directions, achieving an effective balance between privacy, robustness, efficiency, and clinical reliability remains essential for the safe and sustainable deployment of M3T-FedFMs in psychiatric care.

### 4.5 (Challenge 5) infrastructural and human resource barriers

M3T-FedFMs typically contain billions/trillions of parameters and impose substantial computational and memory demands at participating sites, making local training/fine-tuning challenging in many psychiatric settings. The previously described PEFT methods, such as LoRA and adapter-based tuning, can reduce client-side compute and storage requirements by updating only a small subset of parameters rather than the full model backbone. However, PEFT methods alone do not resolve the broader reality that federated clients are heterogeneous in hardware, network bandwidth, and operational capacity (e.g., rural clinics versus academic hospitals). Accordingly, resource-adaptive M3T-FedFM frameworks that explicitly accommodate system heterogeneity (by allowing clients to train scaled-down subnetworks or early-exit variants matched to local compute budgets) can be explored to provide a concrete template for improving participation without requiring uniform resources across sites (84, 85).

Such infrastructural constraints are further compounded by human-capital and operational limitations: in addition to hardware constraints, participation in M3T-FedFMs requires specialized technical expertise that might be unavailable in small psychiatric practices. Specifically, staff must be able to preprocess and format multimodal data (e.g., EHRs, audio recordings, and wearable sensor streams), configure and maintain local model training environments, manage software dependencies, troubleshoot system failures, and ensure compliance with data protection regulations (e.g., HIPAA and GDPR). However, clinical staff typically prioritize patient care and often lack formal training in such ML-focused operations. Subsequently, future research can focus on developing turnkey M3T-FedFM platforms with automated preprocessing pipelines, intuitive interfaces, built-in compliance checks, and targeted model training programs that build internal technical capacity within psychiatric institutions. Without such deliberate efforts to reduce both computational and expertise requirements, M3T-FedFMs risk remaining accessible primarily to well-resourced sites/clinics, thereby reinforcing existing disparities in access to advanced mental health care.

### 4.6 (Challenge 6) data heterogeneity across modalities

In psychiatric applications, clients rarely collect the same set of data modalities. For example, a school may collect only self-reported questionnaires and academic records, while a psychiatric clinic may capture audio from counseling sessions, and hospitals may gather neuroimaging or wearable-derived physiological signals. This heterogeneity creates partially non-overlapping feature spaces across clients in the M3T-FedFMs. Moreover, even within a single data-collecting entity, modality availability can change dynamically: a patient may initially consent to video-based monitoring but later disable camera access, or wearable sensors may intermittently fail to record due to technical or compliance issues. In such settings, clients with incomplete or shifting modality views may contribute noisy, biased, or incompatible updates to the global model, which can in turn cause poor generalization, slower convergence, or even divergence of M3T-FedFMs. A key research direction, therefore, is to develop modality-robust and adaptive learning strategies for M3T-FedFMs that can dynamically align heterogeneous feature spaces across clients, infer missing modalities through cross-modal distillation or generative modeling (86), and ensure stable convergence despite asynchronous or incomplete client contributions, ultimately enabling reliable learning from fragmented and evolving psychiatric data ecosystems.

### 4.7 (Challenge 7) task variability and multi-objective optimization

When deploying M3T-FedFMs for mood disorder analysis, institutions often prioritize different tasks. For example, schools may focus on early detection of at-risk students, clinics on severity classification and treatment response, hospitals on prognosis that integrates comorbid conditions, and personal devices on continuous monitoring and adherence support. These tasks vary not only in output format (classification, risk scores, treatment recommendations, or real-time monitoring) but also in temporal scale (single assessments versus continuous tracking), relevant modalities (EHRs, surveys, or wearable signals), and computational demand. This functional/task diversity challenges the conventional assumption in M3T-FedFMs that all clients share a fixed set of training objectives (85). Although the modular M3T-FedFM architecture supports task-specific heads, these task heads still rely on shared backbone and fusion layers, which are updated based on all client contributions. In this context, since different tasks optimize for different types of signals – for example, speech-based models may prioritize short-term linguistic cues, whereas relapse prediction emphasizes long-term clinical history – their gradients can push the shared backbone in conflicting directions. This task/gradient interference can slow model convergence or bias the M3T-FedFMs toward tasks that are more frequently deployed across clients (e.g., general disorder classification), while degrading their performance on less common but clinically critical tasks, such as suicide risk prediction. Therefore, a key research direction lies in developing task-adaptive strategies for M3T-FedFMs at both the architectural and optimization levels. Specifically, at the architectural level, there is a need to design and rigorously evaluate task-isolated update paths (e.g., separate heads, adapter stacks, or routing gates) that minimize gradient interference while enabling dynamic task-module selection as institutional priorities evolve. Also, At the optimization level, promising directions include (i) developing gradient projection schemes that explicitly constrain conflicting task updates within the shared backbone, (ii) adaptive weighting mechanisms that dynamically adjust task importance during local model fine-tuning (e.g., based on training progress and task performance), and (iii) uncertainty-aware model aggregation strategies that weigh client updates during aggregation according to their confidence or data quality.

### 4.8 (Challenge 8) personalization in embodied and interactive contexts

M3T-FedFMs are expected to operate in interactive psychiatric contexts that require continuous patient monitoring, adaptive feedback, and real-time decision support. In these settings, models must balance generalizability with personalization across patient characteristics, institutional protocols, and clinical contexts. However, the multi-layered nature of personalization in psychiatry complicates the personalization of M3T-FedFMs. Specifically, at the *patient level*, models must capture individual symptom trajectories and behavioral patterns; at the *institutional level*, they must reflect population characteristics, diagnostic practices, and treatment protocols unique to each clinic, school, or hospital; and at the *contextual level*, personalization must account for varying care environments, such as real-time emergency triage versus routine outpatient monitoring. Further complicating this challenge, each of these layers operates on different timescales: patient-level model adaptations may need frequent model updates (e.g., daily, based on mood fluctuations), institutional model adaptations may follow longer cycles (e.g., monthly, aligned with evolving guidelines), and context-aware model adaptations may be triggered by situational shifts (e.g., emergency vs. non-emergency settings). Without explicit coordination across these layers, M3T-FedFMs risk either over-adapting to local patterns (e.g., extended fine-tuning at clients without periodic global aggregation), thereby losing generalizability, or remaining too generic to deliver meaningful therapeutic value (e.g., relying solely on a static global model that fails to reflect evolving patient trajectories or institutional contexts).

To address these aforementioned personalization challenges, two major research directions warrant investigation. First, hierarchical model personalization frameworks with distinct modules for each layer (patient-level prompts, institution-level adapters, context-level gating) updated on appropriate timescales. Second, creating resource-aware model personalization scheduling algorithms that dynamically adjust adaptation frequency and depth based on available computational resources, clinical urgency, and data availability at each client.

## 5 Conclusion

In this work, we aimed to unveil the potential of M3T-FedFMs to transform mood disorder analysis in psychiatry. We illustrated how M3T-FedFMs can leverage diverse data modalities while maintaining data privacy, and highlighted potential applications across clinical, educational, occupational, and personal settings. We also identified key challenges, including HITL design ethical challenges, transparency, modality heterogeneity, task variability, and personalization, which must be addressed for M3T-FedFMs to become clinically viable, and outlined future research directions to overcome these challenges.

## Data Availability

The original contributions presented in the study are included in the article/supplementary material. Further inquiries can be directed to the corresponding author.
